# Supplementary material for: Incorporation of Fly Ash in Flame-Retardant Systems of Biopolyesters
Source: Polymers (Basel). 2023 Jun 21;15(13):2771. doi: 10.3390/polym15132771 (PMC10347290; doi:10.3390/polym15132771)
Supplement: Supplementary file 1 [file polymers-15-02771-s001.zip › polymers-2436762-supplementary.pdf]

polymers

MDPI

Supplementary

Incorporation of Fly Ash in Flame-Retardant Systems of Biopolyesters

Marcos Batistella [1,*], Jean-Claude Roux [1], Nour-Alhoda Masarra [1], Gwenn le Saout [2], Constantinos Xenopoulos [3] and José-Marie Lopez-Cuesta [1]

[1] Polymers Composites and Hybrids, IMT Mines Alès, 30319 Ales, France; jean-claude.roux@mines-ales.fr (J.-C.R.); nour-alhoda.masarra@mines-ales.fr (N.-A.M.); jose-marie.lopez-cuesta@mines-ales.fr (J.-M.L.-C.)
[2] LMGC, IMT Mines Ales, Université Montpellier, CNRS, 30100 Ales, France; gwenn.le-saout@mines-ales.fr
[3] Holcim Innovation Center, 38090 Saint Quentin Fallavier, France; constantinos.xenopoulos@holcim.com
* Correspondence: marcos.batistella@mines-ales.fr; Tel.: +33-46-6785-342

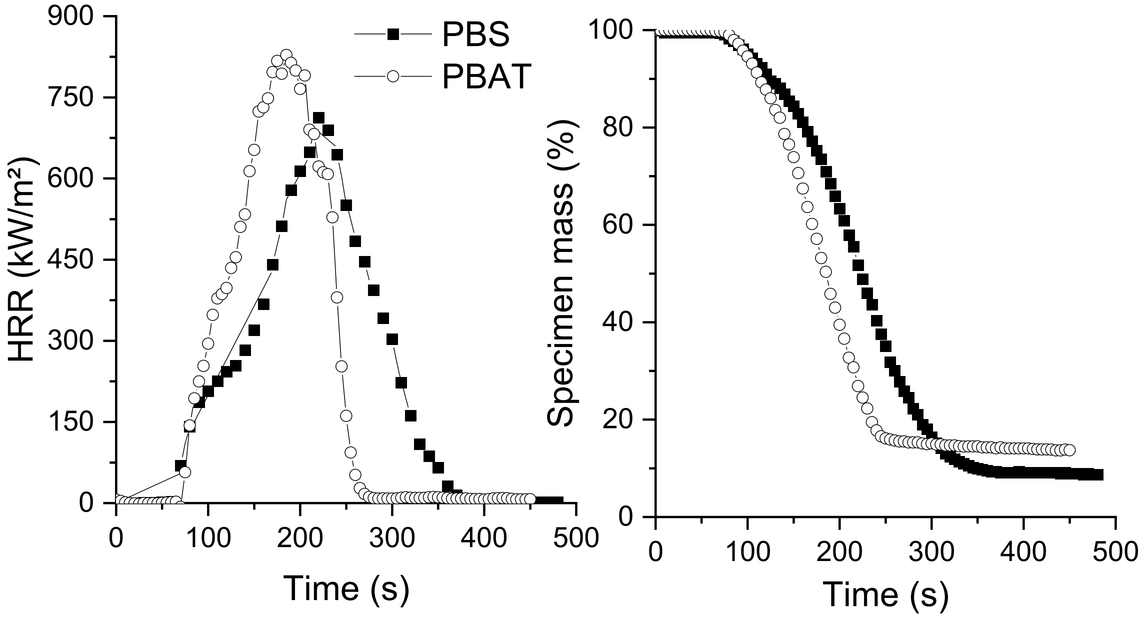

Figure S1. HRR and mass loss curves of PBS and PBAT.

Table S1. Tensile properties of some PBAT/PBS compositions. *Data from technical datasheet.

Formulation	E (MPa)	E (%)	UTS (MPa)
PBS	741±17	20±1.5	37±0.7
PBS-9	1175±24	20±5	24±0.2
PBAT*		560/710	35/45
PBAT-9	150±12	630±17	12±0.15
